# Livestock-Associated MRSA: The Impact on Humans

**DOI:** 10.3390/antibiotics4040521

**Published:** 2015-11-06

**Authors:** Christiane Cuny, Lothar H. Wieler, Wolfgang Witte

**Affiliations:** 1 Robert Koch Institute, Wernigerode Branch, 38855 Wernigerode, Germany; E-Mail: wittew@rki.de; 2 Robert Koch Institute, Main Institute, 13353 Berlin, Germany; E-Mail: wielerl@rki.de

**Keywords:** methicillin-resistant *Staphylococcus aureus*, livestock, zoonotic transmission

## Abstract

During the past 25 years an increase in the prevalence of methicillin-resistant *Staphylococcus aureus* (HA-MRSA) was recorded worldwide. Additionally, MRSA infections may occur outside and independent of hospitals, caused by community associated MRSA (CA-MRSA). In Germany, we found that at least 10% of these sporadic infections are due to livestock-associated MRSA (LA-MRSA), which is initially associated with livestock. The majority of these MRSA cases are attributed to clonal complex CC398. LA-MRSA CC398 colonizes the animals asymptomatically in about half of conventional pig farms. For about 77%–86% of humans with occupational exposure to pigs, nasal carriage has been reported; it can be lost when exposure is interrupted. Among family members living at the same farms, only 4%–5% are colonized. Spread beyond this group of people is less frequent. The prevalence of LA-MRSA in livestock seems to be influenced by farm size, farming systems, usage of disinfectants, and in-feed zinc. LA-MRSA CC398 is able to cause the same kind of infections in humans as *S. aureus* and MRSA in general. It can be introduced to hospitals and cause nosocomial infections such as postoperative surgical site infections, ventilator associated pneumonia, septicemia, and infections after joint replacement. For this reason, screening for MRSA colonization at hospital admittance is recommended for farmers and veterinarians with livestock contacts. Intrahospital dissemination, typical for HA-MRSA in the absence of sufficient hygiene, has only rarely been observed for LA-MRSA to date. The proportion of LA-MRSA among all MRSA from nosocomial infections is about 3% across Germany. In geographical areas with a comparatively high density of conventional farms, LA-MRSA accounts for up to 10% of MRSA from septicemia and 15% of MRSA from wound infections. As known from comparative genome analysis, LA-MRSA has evolved from human-adapted methicillin-susceptible *S. aureus*, and the jump to livestock was obviously associated with several genetic changes. Reversion of the genetic changes and readaptation to humans bears a potential health risk and requires tight surveillance. Although most LA-MRSA (>80%) is resistant to several antibiotics, there are still sufficient treatment options.

## 1. Methicillin-Resistant *Staphylococcus aureus*

The proportion of MRSA among *S. aureus* from nosocomial infections increased considerably from the end of the 1980s until 2000, in nearly all of Europe and worldwide [1]. There are a few countries, such as the Netherlands and the Scandinavian countries, where the consequent implementation of appropriate infection control measures prevented this development [2]. During the past five years the rising trend was halted and even reverted in several European countries [1], which is likely due to the introduction of mandatory surveillance for MRSA bacteremia in some countries [3] and to the region-wide search and follow-up strategies reported in Germany [4,5].

Based on epidemiological criteria, with respect to risk factors associated with treatment and care in nosocomial settings, healthcare-associated MRSA (HA-MRSA) is discriminated from community-associated MRSA (CA-MRSA), which emerged independent of these risk factors [6]. CA-MRSA infections became a public health issue in some parts of the world such as the United States and South America; however, they are comparatively rare in most European countries [7]. HA-MRSA and CA-MRSA are usually differentiated by their structural and functional genomic traits [8]. The observation of hospital-onset infections caused by genotypic CA-MRSA [9] and the establishment of HA-MRSA in the community [10] can blur the epidemiological distinction. MRSA infections in the community can also be caused by livestock-associated MRSA (LA-MRSA). LA-MRSA is initially associated with livestock [11,12,13], and differs from genotypic HA-MRSA and genotypic CA-MRSA in its genomic traits.

The population structure of the species *S. aureus* is largely clonal, *i.e.*, recombinational exchange of parts of the genomes between different strains is rare. Therefore multi locus sequence typing (MLST), which includes allelic profiles of seven housekeeping genes and the resulting sequence types (STs), is a robust framework for tracing the evolutionary origin and spread of MRSA. Isolates that share at least five of the seven MLST alleles are grouped as clonal complexes (CCs) [14]. S*pa* typing [15] is used worldwide as a first-line typing tool. It is based on sequence polymorphisms of the X-region of the *spa* gene and also allows preliminary attribution to CCs.

MRSA evolved from methicillin-susceptible *S. aureus* (MSSA) by acquisition of SSC*mec* elements containing a *mec* gene (*mec*A, more rarely *mec*C), which codes for an additional penicillin binding protein that has low affinity for β-lactam antibiotics and therefore mediates resistance to nearly all compounds from this antibiotic class (besides ceftobiprole and ceftarolin). So far at least 11 different structural types of SCC*mec* are known [16]. Studies leading to our current knowledge on emergence and dissemination of LA-MRSA mainly used these methodological basics. Recently, progress in next generation sequencing has made the rapid and affordable analysis of whole bacterial genomes possible and has facilitated much deeper and more precise insight into the evolution and dissemination of LA-MRSA [17,18,19].

## 2. Host Specificity of *S. aureus* and of MRSA

Nearly 50 years ago, further differentiation of *S. aureus* isolates from humans and a variety of animal species, including livestock, by means of phenotypical characterization, had led to the discrimination of different ecovars (biotypes) of *S. aureus* [20]. Biotypes that could not be attributed to a particular host by the methodology used were also found. Today we know that 87% of *S. aureus* isolates from colonization and infections in humans represent 11 widely disseminated clonal complexes: CC1, CC5, CC8, CC12, CC15, CC22, CC25, CC30, CC45, CC51, and CC121. Clonal complexes CC8, CC15, CC22, CC30, CC45, CC30, CC45, and the rarer clonal complexes CC80 and CC152 are primarily associated with isolates from humans.

MRSA was detected in domestic animals a long time ago [21]. In earl studies the genetic background and antimicrobial resistance of *S. aureus* and MRSA have been associated with host specificity in livestock. This opinion has changed due to studies based on comparative genome analysis. Furthermore, MRSA with low host specificity attributed to CC130 and CC398 emerged. Clonal complexes CC97, CC133, CC522, and clonal lineage ST151 are mainly represented by isolates from ruminants, whereas clonal lineage ST385 is mainly represented by isolates from poultry (for a summary see [2,21,22,23,24]). Comparative analyses of the genomes of MSSA isolates attributed to ST5 from humans and from poultry and of MSSA/MRSA of CC398 revealed that the livestock subpopulations of these clonal complexes originated from ancestral populations in humans [17,25]. On the other hand, human-associated isolates attributed to ST91 obviously originated from ruminants [19]. Companion animals are usually colonized by human-related genotypes [26], although some studies have described colonization factors that determine host specificity [22]. MRSA detection in free-living wild animals in Europe has revealed a low prevalence of genotypes related to livestock and humans.

Genetic changes associated with an adaptation to the animals’ hosts are more related to the accessory genome than to the core genome, and to mobile genetic elements such as prophages and genomic pathogenicity islands [17,25,27,28,29]. Despite these genetic events, LA-MRSA CC398 has retained its capacity to cause infections in humans [30]. Among the LA-MRSA strains described so far, CC398 is most widely disseminated, and we will focus on it particularly in the following text. The second most frequent LA-MRSA clonal lineage is represented by clonal complex CC9, which is disseminated worldwide and seems to be particularly prevalent among various species of livestock in Asia [31]. MRSA attributed to ST5 was recently reported in pigs in the USA [32]. Also, MRSA attributed to CC1 seems to have a low host specificity. It has been identified from infections in humans in many countries and is particularly prevalent among isolates from nosocomial infections in Romania [33]. Containing *luk*-PV (Panton Valentine Leukocidin, PVL), MRSA CC1 emerged in the USA by the end of the 1990s and has been reported in many countries worldwide [34]. MRSA CC1 (PVL negative) was first reported from a cluster of subclinical mastitis in cattle from Hungary [35]. It later on was identified from infections in hospitalized horses [36], as a frequent colonizer of farmed pigs in Italy, and less frequently in pigs from other European countries [37]. The high genetic relatedness of isolates from Italian cattle herds and humans, with respect to typing characteristics and possession of virulence-associated genes, is of particular interest [37].

**Table 1:** Clonal lineages (ST) and clonal complexes (CC) of *S. aureus* observed in humans as well as in animals (references not covered by reviews [21,22,23] are inserted).

Clonal Lineages/Complexes	Human	Livestock						Companion Animals			Marine Mammals	Wildlife			
		Pig	Cattle	Sheep, Goat	Chicken	Turkey	Rabbit	Horse	Dog	Cat		Boar	Ruminants: Red Deer, Roe	Glires: Hare, Rat	Birds
**Originally Known from Humans**															
**CC1**; ST1	MSSA, HA-MRSA, CA-MRSA, PVL+	MSSA ^1^, MRSA [37]	MRSA, subclinical mastitis [35,37]		MSSA			MRSA, [38]							
**CC5**; ST5	MSSA, HA-MRSA, CA-MRSA, PVL+	MRSA [32]			MSSA [25] MRSA			MRSA [38]							
**CC5**	HA-MRSA (Europe)							MRSA [38]	MRSA						[39]
**CC7**; ST7	MSSA, MRSA	MSSA				MSSA ^1^									
**CC8**; ST8	MSSA, HA-MRSA, CA-MRSA, PVL+	CA-MRSA, PVL+ (USA) ^2^						MRSA (Northern America, Europe)		MRSA, PVL+ ^1^	MRSA [39]			MRSA [39]	
**CC8**; ST239	HA-MRSA				MRSA (Belgium)										
**CC8**; ST254	HA-MRSA							MRSA (Central Europe)							
**CC15**; ST15	MSSA, HA-MRSA ^2^								MSSA ^1^						
**CC22**; ST22	MSSA, HA-MRSA, CA-MRSA, PVL+							MRSA ^1^ Own observation	MRSA [40]	MRSA [40]					
**CC30**; ST30	MSSA, CA-MRSA, PVL+	MSSA (Germany)													
**CC30**; ST36	HA-MRSA								HA-MRSA	HA-MRSA (New Zealand)					
**CC45**; ST45	MSSA, HA-MRSA								MSSA	MSSA					
**CC59**; ST59	MSSA, HA-MRSA, CA-MRSA	MRSA [31]			MRSA (Asia)										
**CC398**; ST398 human subpopulation	MSSA, MRSA, PVL+ [17,41]														
**CC398**; ST398 Animal sub-population, LA-MRSA	MSSA, LA-MRSA ^3^	MSSA, MRSA	MRSA	MRSA [42,43]	MRSA	MRSA	MRSA [44,45]	MRSA ^4^	LA-MRSA	LA-MRSA					
**Originally Known from Animals**															
**CC9**; ST9	MSSA, LA-MRSA ^4^	MSSA, LA-MRSA	LA-MRSA ^1^		LA-MRSA										
**CC97**; ST97	MSSA ^1^, LA-MRSA ^1^	MSSA [19]	MSSA, LA-MRSA ^1^ [19]												
**CC130**; ST130	MRSA ^1^[37,46]		MRSA [47]	MRSA [47]				MRSA ^1^ (own observation)	MRSA [48]	MRSA [48]	MRSA [47]		MRSA [46,49] MSSA [49]	MRSA [46,47,49]	MRSA [50]
ST425	MRSA [51]		MRSA [51]									MSSA [52]			
**CC133**				MSSA [53,54]								MSSA [52]			
**CC522**				MSSA [53,54]											

^1^ rarely reported; ^2^ own rare observation; ^3^ frequent colonizers of humans with professional exposure to livestock; infections are altogether rarer so far; ^4^ equine hospital-associated subpopulation; HA-MRSA: hospital-acquired MRSA, CA-MRSA: community-acquired MRSA, LA-MRSA: livestock-associated MRSA, PVL: Panton-Valentine leukocidin.

During the past five years, MRSA attributed to CC130, which contains the homologue *mec*C instead of *mec*A, gained attention. Host specificity for this clonal complex also is limited. Isolates attributed to CC130 have been reported from domestic animals, especially cattle, sheep, goats, dogs, and cats, as well as from wildlife such as roe deer, chamois (MSSA), brown rats, seals [47,48,50,55], and even from wildlife such as hares (captive mara) [56]. Interestingly, MRSA CC130 was observed in wildlife as well as in domestic ruminants sharing the same habitat, suggesting mutual exchange [57]. Although MRSA CC130 is also able to cause infections in humans [55], these cases are rare so far [46]. The homologue *mec*C is also present in MRSA attributed to ST425, which emerged as a cause of mastitis in dairy cattle and was also identified in human infections [51]. Furthermore, MSSA attributed to ST425 was reported from nasal colonization of wild boars besides the isolates attributed to ST133 [52].

When introduced to veterinary clinics, epidemic HA-MRSA can cause clusters of nosocomial infections as was reported for MRSA ST22 in small animal departments [58,59]. MRSA ST8, spa type t064, which was first observed in Canadian equine clinics and has since been reported in other parts of the world, seems to originate from a hospital-associated ST8 subpopulation [60]. Interestingly, MRSA with these typing characteristics was also obtained from marine mammals (dolphins, orcas, beluga whales, and walruses) in a Canadian marine park [61]. MRSA ST254, observed in Northern Germany and in the United Kingdom, was reported in infection clusters in equine hospitals and shares genetic characteristics with HA-MRSA [36,62]. MRSA from humans may also spread to wildlife, as suggested by the report on an isolate exhibiting spa type t008 (CC8) in a cottontail and of isolates with spa type t002 (CC5) in lesser yellowlegs in Iowa [39]. Data on *S. aureus*/MRSA in animal species are compiled in Table 1. We should, however, note that a number of these data points originate from cross-sectional studies, and that permanent or at least intermittent MRSA colonization remains to be shown.

## 3. LA-MRSA in Livestock Animals

The first communication on LA-MRSA CC398 colonizing conventionally raised pigs was followed by several reports from European countries with pronounced conventional farming such as the Netherlands, Denmark, Germany, France, Italy (for summary, see [63]), and later on from North America [32], Northern Africa [64], Asia [31], and Australia [65]. Initially this concerned pigs, later veal calves [66] and poultry [67]. LA-MRSA CC398 has also been reported in dairy cattle [68] and in turkeys [69]. Infections in livestock caused by LA-MRSA CC398 are very rare [70]. The emergence of LA-MRSA in livestock seems to correlate with farm size [71,72], farming systems (conventional *vs.* alternative [73,74]), usage of disinfectants, and in-feed zinc [75]. The spread of LA-MRSA between farms is often mediated by animal trading, namely of piglets that are sold by specialized producers [76]. As is to be expected, and can hardly be prevented, raw meat products can be contaminated during processing. When 2217 meat samples were checked in the Netherlands, MRSA contamination was found for 10.7% of pork, 15.2% of beef, 15.2% of veal, 6.2% of lamb, and 35.3% of turkey meat [77]. A German study has shown this contamination in 2.8% of pork end products [78]. In 2010, a contamination rate of 32% was reported in turkey meat [79]. Similar contamination frequencies were reported from Canada and the Unites States of America [80,81], as well as from Taiwan [82]. Involvement of MSSA/MRSA CC398 in food intoxication has not been reported to date, and isolates attributed to this clonal complex only rarely seem to contain enterotoxin genes [83,84]. This may change as MRSA CC398 can reacquire the immune evasion gene cluster (IEC), which is contained by prophages and is typical for *S. aureus* in humans. Particular types of IEC can contain *sea* or *sep* [85]. IEC was found in 19% of LA-MRSA CC398 infections in humans (*n* = 99); only one of these isolates contained *sea* [41]. IEC-containing isolates from pigs have not been identified so far; however, it is more frequent among isolates attributed to the equine clinic-associated subpopulation originating from horses and their caretakers. One of these isolates was found to contain *sea* [41]. The finding of LA-MRSA CC398 in tank milk suggests udder colonization and possibly cases of subclinical mastitis in dairy cattle in Germany [86]. LA-MRSA CC398, meanwhile, has also arrived in industrial rabbit farms [44], and has been observed in a pet rabbit [45].

## 4. LA-MRSA CC398 in Other Animals besides Livestock

At farms in Belgium where pigs were colonized with LA-MRSACC398 other animals such as goats, cats, dogs, mice, and rats as well as humans residing on these farms were also found to be colonized [43]. MRSA CC398 in particular emerged as a nosocomial pathogen in equine clinics about 10 years ago in Austria [36], the Netherlands [87], Belgium [88], and Switzerland [89]. Meanwhile, it comprises most of the MRSA isolates from nosocomial infections in horses in Germany [90]. MRSA CC398 associated with equine clinics represents a separate subpopulation [91]. High proportions of MRSA CC398 were also reported for isolates from wound samples from other companion animals such as cats and dogs in Germany [90,92].

## 5. Transmission of LA-MRSA to Humans

Transmission of *S. aureus* between hosts is primarily mediated by physical contacts. Dust in stables with MRSA-colonized pigs is heavily contaminated [93]. Therefore it seems likely that colonization of humans working in these areas takes place by inhalation of MRSA-contaminated dust [94]. Nasal colonization has been found in 77%–86% of humans working in MRSA-positive stables [95,96]. The extension of colonization seems to be dependent upon the duration of exposure and upon the intensity of animal contacts [97]. For a considerable proportion of the farmers, the MRSA colonization continued when the stable exposure was interrupted by holidays [98,99]. Other persons residing on these farms (e.g., household members) were less frequently colonized (4%–5%, [95]).

A comparative longitudinal study performed in Belgium, Denmark, and the Netherlands revealed that pig contact was the most important determinant for MRSA carriage among household members of farmers (Belgium 29%, Denmark 0%, Netherlands 6%). The increased MRSA carriage rate observed among household members from Belgium seems to be linked to country-specific differences in pig exposure [100]. Another study performed in the Netherlands came to the conclusion that working with sows and living with an MRSA-positive pig farmer were significant determinant for MRSA carriage of household members [101]. In the Netherlands a national program for reduction of antibiotic use in animal farming was initiated in 2010. In Germany a longitudinal study of MRSA colonization of pigs, humans working on pig farms, and their household members has shown that the 44% reduction of antibiotic use at the farms enrolled in this study was associated with declining MRSA prevalence in pigs and LA-MRSA in humans, independent of animal contact [102]. In Taiwan nasal carriage of LA-MRSA ST9 among pigs was found to be higher in large farms than in smaller ones (34% *vs.* 7%); this was mirrored by the carriage rates of humans in occupational contact with pigs (36.8% *vs.* 9.1%) [103].

Nasal colonization with LA-MRSA was also found in slaughterhouse workers [104,105], in veterinarians in Germany [95,106], and in veterinarians in Belgium [107], where veterinary attendance of livestock was identified as a major risk factor. Household members of veterinarians were also found to be colonized [95,108]. The use of whole-genome maps for studying LA-MRSA colonization in families of veterinarians indicated possible transmission of LA-MRSA between humans [109].

Most of these data were obtained from studies on conventional farms. LA-MRSA CC398 was not found in pigs and humans at organic farms in Germany [73], and was clearly less prevalent in pigs at organic farms in comparison to conventional ones in the Netherlands [74]. Dissemination of LA-MRSA among humans beyond farms seems to be rare, as suggested by a study on pupils of a school in a German area with a high density of pig farms in the northwest of Germany [95]. Nevertheless, as demonstrated through screening of patients at hospital admission, the prevalence of LA-MRSA is considerably higher in northwest Germany than in all of Germany [110]. This corresponds to observations in the Netherlands, where livestock density was identified as a risk factor for livestock-associated methicillin-resistant *Staphylococcus aureus* [111]. Emission of LA-MRSA in exhausted air from pig stables has been shown by several studies, and has been found in air up to 350 m downwind from stables and up to 500m distant on the soil surface [112]. LA-MRSA has also been found in manure from chicken farms and in soil fertilized with this manure [113]. In this context, demonstration of LA-MRSA in fecal samples from rooks in Austria is of interest [114]. The question of whether living in close vicinity to conventional livestock farms bears an increased risk for colonization with LA-MRSA needs further elucidation. A study in Lower Saxony in Germany found nasal colonization with LA-MRSA CC398 in about 1% of humans who have their home near livestock farms [115]. Of particular interest are results from an extensive epidemiological study in Pennsylvania, USA, where skin and soft tissue infections with MRSA were found more frequently in humans living close to fields that were fertilized with manure from conventional farms. Unfortunately the study was limited due to lack of typing of a sufficient number of MRSA isolates both of animal (manure) and human origin [116]. As LA-MRSA CC398 was also reported from dogs and cats, transmission to humans seems likely and should be a reminder of the importance of basic hygiene in households [117].

It was reasonable to assume that human-to-human transmission of LA-MRSA CC398 occurs more rarely. There are, however, recent data from Spain [118] and from Germany [119] on LA-MRSA infections in humans who had no contact with animals. In the Netherlands it was observed that in 15% of all LA-MRSA CC398 human cases, the persons had not been in direct contact with pigs or veal calves [120].

Besides human-to-human transmission and environmental exposure, LA-MRSA may also be acquired by handling contaminated meat products. This could be particularly applicable to professional food handlers, as a study in the Netherlands revealed a statistically significant higher risk of acquisition of LA-MRSA for persons with regular consumption of poultry [121].

## 6. LA-MRSA Infections in Humans

LA-MRSA CC398 possesses the same virulence potential as *S. aureus* from humans in general and is associated with the same manifold clinical pictures. Outside of the hospital setting, these are mainly infections of skin and soft tissue that require surgical interventions. Affected patients are primarily persons with occupational exposure to livestock and occasional contact with them. LA-MRSA represents about 13% of MRSA-linked severe skin and soft tissue infections [13]. Although the incidence of these infections in Germany is not known, they seem to occur rarely.

LA-MRSA can enter hospitals either via patients who suffer from infections caused by these bacteria who need appropriate treatment or by patients with nasal colonization. The latter can lead to nosocomial infections such as surgical site infections, infections after joint arthroplasty, ventilator associated pneumonia, or septicemia [30,122]. A survey on MRSA colonization at hospital admission in the south of Brandenburg federal state of Germany, an area with a low density of livestock farms, reported colonization with LA-MRSA CC398 in 0.08% among the 13,855 investigated individuals [123]. In the Ems-Dollart area of North Rhine Westphalia federal state, which has a substantial density of livestock farms, the proportion of LA-MRSA CC398 among all MRSA detected by screening at hospital admission (altogether 1.6% of all individuals) increased from 14% in 2008 to 23% in 2011. Correspondingly the proportion of LA-MRSA among MRSA from wound infections increased from 7% to 10% during the same period of time [111]. The proportion of LA-MRSA among MRSA from septicemia in this area is about 10%, whereas it is substantially lower (1.8%) in all North Rhine Westphalia [124], which corresponds to the proportion reported for all Germany [13].

Proportions of LA-MRSA among all MRSA isolates from colonization and infections in humans have to be assessed in relation to the overall MRSA prevalence. They are, of course, higher in countries where the prevalence of HA-MRSA is low, such as the Netherlands. Among patients screened at hospital admission, 9.7% were found to be positive for MRSA; of these, 78% were LA-MRSA and 22% non-LA-MRSA [125].

Human-to-human transmission of LA-MRSA CC398 in hospitals leading to a small cluster of infections has been observed [126], but it is obviously much rarer in comparison to HA-MRSA [127].

LA-MRSA CC398 is capable of being established in a hospital, as shown by whole genome sequencing-based phylogenetic analysis for a subclade represented by isolates from the environment and from newborns in a Scottish hospital [128].

In the case of infections that require antibiotic treatment, the current antibiotic resistance profile of LA-MRSA leaves sufficient alternatives. Usually LA-MRSA is resistant to β-lactams, macrolides, lincosamides, streptogramines, tetracyclines, and in part to fluoroquinolones as well as to cotrimoxazole. It is susceptible to glycopeptides, daptomycin, tigecyclin, rifampicin, fusidic acid, fosfomycin, and with few exceptions also to linezolid. Resistance to linezolid demands alertness. Besides mutations in 23S rRNA and in ribosomal proteins, linezolid resistance can be transferred by acquisition of a plasmid harboring the transferrable *cfr* gene that mediates multi-resistance against linezolid, lincosamides, fenicols, and pleuromutilins by dimethylation of 23S rRNA [129]. Thus, selective pressure in favor of spread can be exerted by the use of linezolid in human medicine and of florfenicol and tiamulin in veterinary medicine [130]. *Cfr* first became known in coagulase-negative staphylococci from livestock in Europe [131] and more recently has been reported in China [132]. In general, linezolid resistance is still rare in CNS from infections in humans [133], but clusters of nosocomial infections with *cfr* containing *S. epidermidis* have been reported [134]. Only singular isolates of LA-MRSA of animal origin containing *cfr* have been identified in Europe [135], but they seem to be more frequent in China [132]. Linezolid resistance is still rare in MRSA of human origin [136]. There has been only one report of a linezolid-resistant human isolate, beyond the emergence of an epidemic of *cfr* containing HA-MRSA in a Madrid hospital [137].

## 7. Outlook

**Figure 1:**
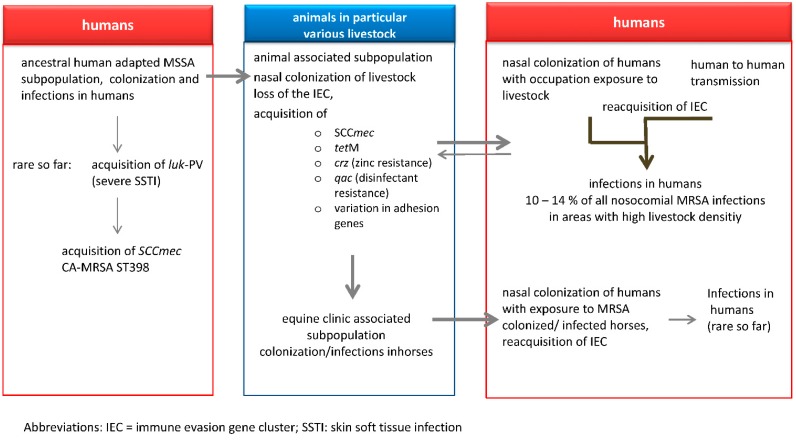
*Staphylococcus aureus* CC398 in humans and in animals.

Particular attention must be paid to early recognition of a scenario in which LA-MRSA could readapt to humans and be retransmitted to animals, where it can become a frequent colonizer and then can again transmit to humans and have a pronounced capacity to spread. *S. aureus* variants adapted to humans usually contain the immune evasion gene cluster IEC [84], which was lost with the evolution of LA-MRSA from its human-adapted ancestor [17]. The finding of a considerable portion of LA-MRSA containing IEC among MRSA infections in humans signals readaptation to humans. Observation of IEC among isolates of the equine clinic-associated subpopulation of MRSA CC398 indicates acquisition of IEC when colonizing veterinary staff and retransfer to horses [41]. Possession of IEC is not a general prerequisite for the capacity of LA-MRSA CC398 to cause invasive infections in humans. As suggested by McCarthy and Lindsay [138], each lineage of *S. aureus* evades host immune responses by a variety of mechanisms. The exact role of IEC in permanent colonization and infection in humans remains to be shown. Although staphylokinase does not seem to be essential for the first step of nasal colonization in humans, it might be important for maintaining it [139]. Two particularly virulent groups of MSSA of human origin are of interest for reflecting on the readaptation of LA-MRSA CC398 to humans: (i) those exhibiting *spa*-type t571; and (ii) those containing *luk*-PV genes, which code for the Panton-Valentine leukocidin. PVL is associated with invasiveness, in particular with deep-seated infections of skin and soft tissue. MSSA C398, t571, attributed to the human subpopulation [17,27], obviously possesses a substantial virulence potential with respect to septicemia and severe infection of skin and soft tissue [27]. Clusters of infections have been reported in the New York area as well as in France and Belgium; in Germany they are rare so far [140]. Although there are important data from comparative genomic analysis [23], it remains to be shown which genome alterations render these isolates particularly virulent. MSSA CC398 containing *luk*-PV was identified first in China [141] and later on in Scandinavian countries [142]. There are also MRSA CC398 *luk*-PV infections that emerge sporadically in Germany. They have been attributed to the ancestral subpopulation and do not represent LA-MRSA that acquired *luk*-PV [41]. Our current knowledge of the transmission of *S. aureus* from humans to animals is illustrated in Figure 1. The first observations of human-to-human transmission of LA-MRSA require further genomics-based analysis of the isolates involved and targeted surveillance in close cooperation with human and veterinary institutions.
